# Dislocated 4-haptic intraocular lens rescue with Gore-tex suture scleral re-fixation

**DOI:** 10.1186/s40942-024-00562-4

**Published:** 2024-07-07

**Authors:** Luke Michaels, George Moussa, Hadi Ziaei, Andrew Davies

**Affiliations:** 1 Lancashire Eye Centre, Lancashire Teaching Hospitals NHS Foundation Trust, Preston Rd, Chorley, PR7 1PP UK; 2 Manchester Royal Eye Hospital, Oxford Road, Manchester, M13 9WL UK; 3 University of Birmingham, Edgbaston, Birmingham, B15 2TT UK

**Keywords:** Dislocated, Subluxed, Intraocular lens, Akreos adapt, Gore-tex

## Abstract

**Background:**

Dislocated IOL exchange conventionally involves manipulation within the anterior chamber which risks secondary injury to anterior chamber structures. We describe and evaluate a 4-haptic IOL rescue technique that avoids entering the anterior chamber and thus minimizes post operative inflammation, astigmatism and recovery time relative to conventional IOL explantation and replacement techniques.

**Methods:**

Retrospective, non-randomized, interventional study of all patients undergoing 4-haptic IOL rescue performed by two independent vitreoretinal surgeons at a single UK centre over two years. Surgical technique: A limited peritomy is performed with four 25-gauge scleral ports placed to enable use of two forceps, an infusion and a chandelier. A further four 27-gauge sclerotomies are symmetrically placed on the nasal and temporal sclera at 3 mm from the limbus with a 5 mm vertical separation on either side. A pars plana vitrectomy is performed followed by chandelier illuminated, bimanual cleaning of the dislocated IOL using 27-gauge serrated forceps. Gore-tex sutures are threaded through the IOL islets within the vitreous cavity and externalized through the sclerotomies for scleral re-fixation followed by conjunctival closure.

**Results:**

Seven patients underwent IOL recycling with Gore-Tex suture scleral re-fixation. All procedures were successful in repositioning the IOLs, with all patients satisfied with post-operative outcome. Mean (standard deviation) time to IOL dislocation was 13 (3) years. Median visual acuity significantly improved post-operatively from 0.85 logMAR (Interquartile Range [IQR]: 0.2–2.1) to 0.07 (0.02–0.60) logMAR (*p* = 0.02). No significant post-operative complications were noted apart from persistent cystoid macular oedema in one patient non-compliant with post-operative treatment.

**Conclusions:**

Transscleral refixation using Gore-Tex suture is an effective, safe and practical approach in the management of dislocated 4-piece IOLs.

**Supplementary Information:**

The online version contains supplementary material available at 10.1186/s40942-024-00562-4.

## Background

Recent years have seen the development and refinement of multiple techniques for the refixation and replacement of dislocated intraocular lenses (IOLs) [1–3]. Posterior chamber transscleral suture fixation techniques are often used where there is no residual capsular support for secondary IOLs and where anterior chamber IOL fixation is not possible or undesirable due to lack of iris support or due to risks of corneal decompensation.

IOL dislocation usually occurs many years after initial IOL implantation and dislocated IOLs can lead to disruption of the anterior chamber or damage to the corneal endothelium. Reduced corneal endothelial cell counts, as seen in elderly patients, is a poor prognostic factor of IOL exchange and therefore avoiding manipulation within the anterior segment by recycling the posterior chamber IOL is desirable to minimise damage to the cornea, iris, or anterior chamber [4]. 

There may be additional complicated scenarios where an anterior chamber approach is not optimum, such as in the presence of a trabeculectomy or glaucoma drainage device. This is a potentially common scenario in cases of pseudoexfoliation (PXF) glaucoma, particularly as PXF is also a risk factor for IOL dislocation [5–7]. Creation of a large corneal wound in an anterior approach to explant and replace an IOL also risks post operative hypotony, astigmatism and delays post-operative recovery [8, 9]. Furthermore, IOL exchange has also been shown to produce more frequent complications such as choroidal effusion, cystoid macular edema, epiretinal membrane and retinal detachment when compared to re-fixation [4, 10]. 

Primary implantation of 4-haptic IOLs, such as such as the Akreos Adapt AO (Bausch & Lomb, Bridgewater, NJ), is common practice at the time of cataract surgery in our region and across the UK [11, 12]. Four-haptic IOLs with perforated haptics or islets are ideal for 4-point scleral fixation as they allow for sutures to be threaded through the IOL islets to suspend the lens.

In this paper we describe and evaluate a 4-haptic IOL rescue technique that avoids entering the anterior chamber which thus minimizes post operative inflammation, astigmatism and recovery time relative to conventional IOL explantation and replacement techniques.

## Methods

We conducted a retrospective, non-randomised, single-centre (Lancashire Eye Centre, Chorley and South Ribble Hospital, Lancashire Teaching Hospitals), interventional study on consecutive dislocated 4-haptic IOLs. Surgery was performed by two independent vitreoretinal surgeons (authors Andrew Davies and George Moussa) over two years (April 2021 to April 2023).

This study adhered to the principles of the Declaration of Helsinki and was registered and approved by our local clinical effectiveness team (Clinical Effectiveness Department, Chorley and South Ribble Hospital, Lancashire Teaching Hospitals). As this was a retrospective anonymized study, and according to our local protocol from our Clinical Effectiveness Department, and as per national guidelines from the National Code of Clinical Research, and the Health Research Authority (HRA), this study has ethical approval exemption. All procedures were completed prior to the design of this study. Patients were diagnosed and treated according to local guidelines and agreements and written consent from patients was acquired prior to all procedures as clinically indicated. Patients had planned follow up appointments at day 1, 2 weeks and 6 weeks post-operatively.

The data were extracted from the electronic patient records and anonymised for analysis.

Data collection included:

I. Baseline demographics and ocular characteristics: Age, pre-operative visual acuity (VA), intraocular pressure (IOP), years since initial IOL insertion and co-pathology.
II. Intraoperative characteristics: Laterality, sclerotomy location and complications.
III. Post-operative characteristics at day 1, 2 weeks, 6 weeks and final visit: post-operative VA, IOP, IOL centration, complications and anterior chamber activity using the Standardization of Uveitis Nomenclature (SUN) system [13].

Pre-operative and post-operative VA were documented as logMAR units from EDTRS charts. For logMAR VA outcomes, pre-operative VA and post-operative VA were defined as the better of corrected distance VA (CDVA), uncorrected distance VA (UDVA) or pinhole VA (PHVA) as reported in the national ophthalmology database (NOD) audit [14]. Non-numeric values were converted to logMAR equivalents as follows: count fingers (CF) = 2.1 logMAR, hand movement = 2.4 logMAR, light perception = 2.7 logMAR, and no light perception = 3.0 logMAR [15]. 

### Statistical analysis

The statistical analysis was completed with IBM SPSS Statistics software (version 29; Armonk, NY: IBM Corp). Statistical significance was defined as *p* < 0.05. Prior to analysis, continuous variables were assessed using the Shapiro-Wilk test for normality. A Wilcoxon signed ranked test was used to compare paired samples from two groups where the data were non-normally distributed.

### Surgical technique

**Fig. 1:**
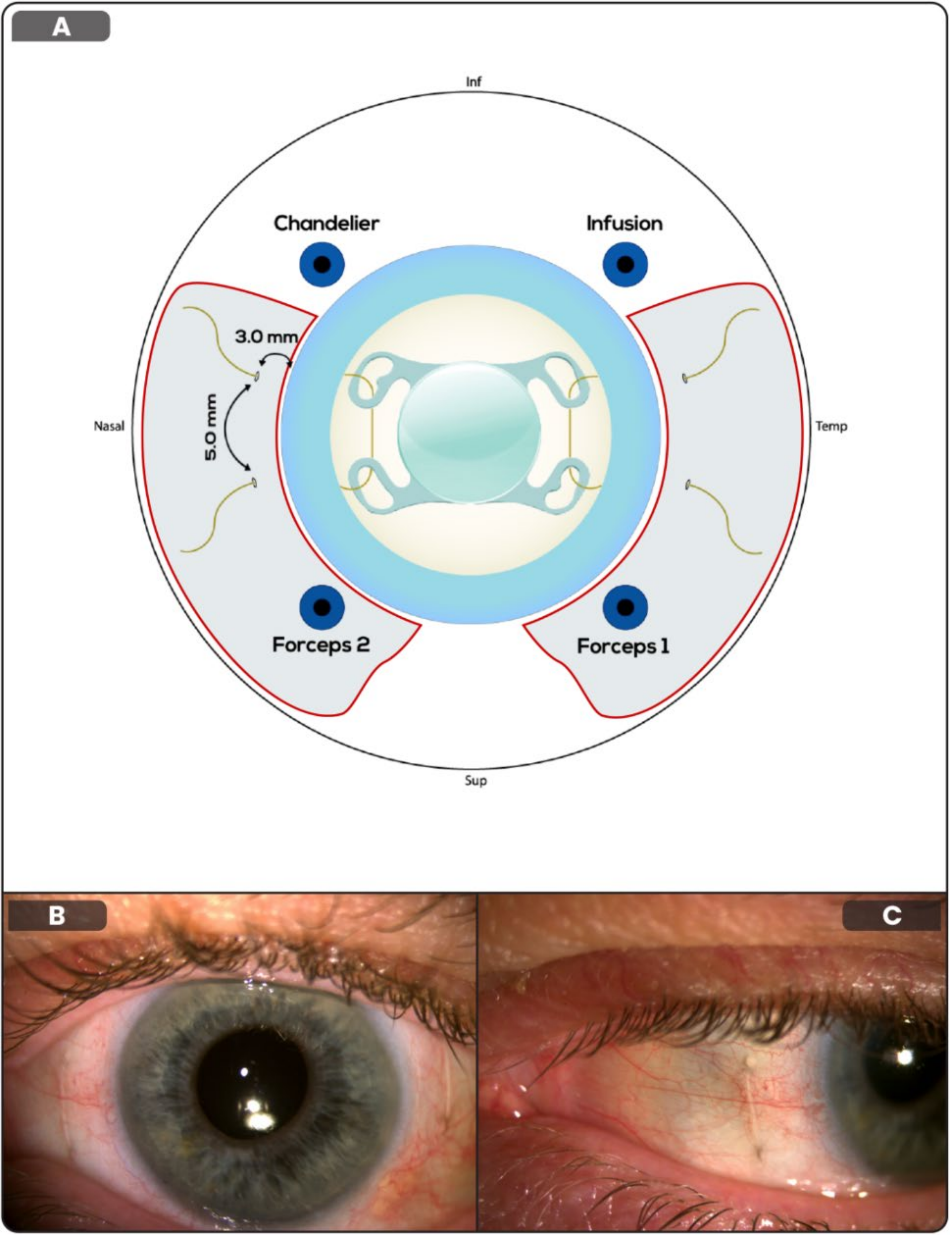
Diagrammatic representation of surgical technique. B and C: photos of Gore-Tex sutures in situ at six weeks post-operatively

Our 4-haptic IOL rescue scleral re-fixation technique that avoids entering the anterior chamber is detailed in Fig. 1 and a supplementary video (Additional File 1) and further described below:

Limited nasal and temporal peritomies are performed to allow for adequate scleral exposure. Three-standard 25-gauge pars plana vitrectomy (PPV) valved sclerotomy ports are placed with two superior ports at the 10 and 2 o’clock positions whilst the infusion port is placed at 8 or 4 o’clock for the right or left eye respectively. To enable bimanual posterior segment manipulation with forceps, a 4th valved sclerotomy port is required for chandelier assisted intraocular illumination and is placed inferonasally. This setup allows for the dislocated IOL to be grasped with serrated jaw forceps and for a CV-8 Gore-Tex (W.L. Gore & Associates, Newark, DE, USA) suture to be threaded through the IOL haptics in an anterior to posterior fashion. This ensures that the IOL rests within the sulcus plane and the inferiorly threaded suture minimizes iris chaffing. Afterwards, four further 27-gauge sclerotomies are placed symmetrically at 2.5–3 mm from the limbus with a 5 mm vertical separation. This technique evolved from a 6-sclerotomy technique to an 8-sclerotomy technique as it was the authors experience that in the presence of 8-sclerotomies (with 4 ports in situ), surgical time is reduced due to the optimal positioning of the 27-gauge sclerotomies which only need to be entered once with 27-gauge serrated jaw forceps. This minimizes multiple entries through the sclerotomies and thus trauma to the sclerotomies which may result in post-operative leak and hypotony. 27-gauge serrated jaw forceps were found to be the easiest forceps to use for both IOL and suture manipulation.

A core and peripheral shave vitrectomy are performed to free the IOL and an indented search performed to examine for any retinal breaks. In the event of dislocation of the entire capsular bag-lens complex, the capsule can be easily removed from the IOL bimanually using two serrated forceps and utilizing illumination form the inferonasal chandelier. Perfluorocarbon liquid can be placed to protect the macula at the surgeon’s discretion. The suture is passed along with a 27-gauge forceps through the 1st sclerotomy. Whilst grasping the IOL with another forceps, the suture is threaded antero-posteriorly through the first IOL islet. The first forceps is then passed through the adjacent sclerotomy to thread the suture through the second islet and then externalized using a handshake technique. The IOL should now be anterior enough to perform the rest of the surgery with the anterior segment microscope. This bimanual technique allows for atraumatic swift threading of the Gore-Tex suture through the islets and externalization of the suture on the nasal and temporal side. Three-one-one surgical knots are tied and buried within the sclerotomies to enhance the sclerotomy seal and minimize hypotony. The sclerotomy ports are removed and sutured to prevent hypotony. Conjunctival closure can then take place to finish the procedure.

## Results

A total of seven eyes of seven patients were included in this study. There were 3/7 males and 5/7 right eyes. The mean age was 56 years old (standard deviation [SD]: 16). All patients were found to have a dislocated 4-haptic Akreos Adapt AO (Bausch & Lomb). The mean number of years since IOL implantation was 13 (3) years.

The baseline demographic and ocular characteristics along with clinical outcomes can be seen in Table 1(end of document).

**Table 1:** Demographic and ocular characteristics with post-operative clinical outcomes

Patient Number	Eye laterality	Age	Co-pathology/contributing factors	Time since IOL implantation (years)	Pre-op IOP (mmHg)	Post-op IOP	Pre-op VA – (logMAR)	Post-op VA at Final visit (logMAR)
1	Right	55	Right pars plana vitrectomy for retinal detachment	14	30	21	1.5	0.2
2	Left	67	Blunt trauma to left eye, corneal guttata	17	18	21	2.1	0.12
3	Right	31	Retinitis pigmentosa, blunt trauma	13	9	10	2.7	0.6
4	Left	51	Retinitis pigmentosa	16	15	16	0.1	0.02
5	Right	83	PXF, PXF glaucoma	11	18	19	0.2	0.02
6	Right	56	Refractive laser surgery, high myopia, previous right pars plana vitrectomy for retinal detachment, corneal guttata	10	15	9	1.42	0.1
7	Right	55	Systemic features suggestive of undiagnosed genetic disorder (short stature, shallow orbit, learning difficulties)	12	11	10	2.1	0.9

All surgeries were performed according to the technique detailed above. Almost all (6/7) patients had sclerotomies sited at 3 mm from the corneal limbus and one patient had sclerotomies sited at 2.5 mm from the limbus due to their eye’s short axial length. No intraoperative complications were noted.

Surgical repositioning using a 4-haptic IOL rescue technique was successful in achieving a well centred scleral re-fixated IOL in all patients. All patients were satisfied with the surgical outcome.

**Fig. 2:**
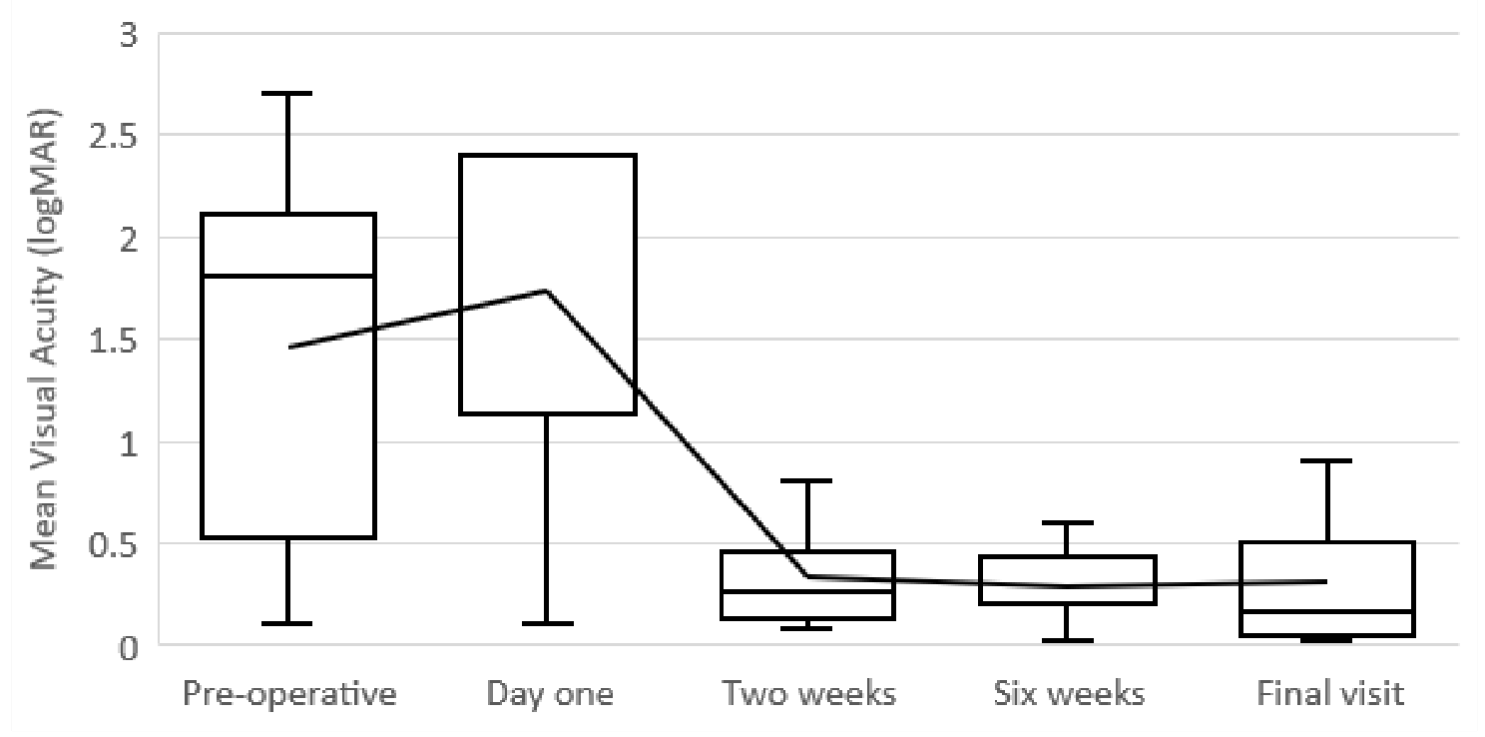
Mean visual acuity outcomes at different time points post Gore-Tex suture scleral re-fixation. Post operative anterior chamber activity did not have any clinically significant repercussions. Day 1 anterior chamber activity ranged from 0.5 + to 1 + cells, 1 + to 2 + cells at 2 weeks and at 6 weeks anterior chamber activity was 0 + in all patients

The median (interquartile range [IQR]) pre-operative VA was 0.85 logMAR (0.2–2.1). The median post-operative VA at day 1, 2 weeks and 6 weeks were 2.40 (0.55–2.40), 0.21 (0.10–0.50), 0.20 (0.10–0.50) logMAR respectively. VA recorded at the final visit significantly improved from preoperatively from 0.85 (0.2–2.1) to 0.07 (0.02–0.60) logMAR (*p* = 0.02) with all patients included (Fig. 2).

### Post-operative complications

Two (29%) patients developed post-operative cystoid macular edema (CME). One of these was also a steroid responder but CME resolved on completion of treatment. The other patient (patient 7) had learning difficulties and was not compliant with topical therapy for CME. At her last review due to ongoing CME the patient was treated with orbital floor steroid and is undergoing further review. One patient (patient 3), who had a background of retinitis pigmentosa, did not attend for final review as patient reported he was happy with his vision and that visually he was back to his baseline. Another patient (patient 5) had labile pressures post-operatively due to PXF glaucoma which was adequately controlled with topical therapy and his IOP control had returned to baseline at the final visit.

There was no significant change in pre- and post-operative IOP (*p* = 0.80). Median (IQR) pre-operative IOP was 15 (11–18) mmHg. The median post-operative IOP at day 1 was 4 (3-7.5) mmHg, 2 weeks, 15 (12–35) mmHg and at 6 weeks was 16 (10–21) mmHg. At day 1, three patients had hypotony (defined as < 5mmHg) which corrected itself within two weeks without further intervention or change to the post-operative plan.

At 2 weeks, patients with IOP > 24mmHg were started on topical antihypertensives for the duration of their four-week post-operative topical steroid regime at 4 weeks. Therefore, no patients by 6 weeks required topical antihypertensive drop therapy.

## Discussion

In this manuscript we report on a flapless transcleral 4-point IOL re-fixation technique using Gore-Tex suture for a 4-haptic IOL. The main advantage of this technique is that no incisions are made in the cornea, and there is no manipulation within the anterior chamber. To our knowledge this is the first description of a such a technique. This is a significant modification of previously described methods for scleral fixation of 4-haptic IOLs such as the Akreos Adapt AO (Bausch & Lomb) and can be applied for use in all 4-haptic IOLs including modern hydrophobic 4-haptic IOLs such as the Micropure (BVI) [16, 17]. 

Comparison of the VA results in eyes treated with PPV and lens explantation relative to repositioning/refixation have been shown to be similar [18, 19]. Our results reflect these findings with all patients achieving an improvement in their VA. A noteworthy advantage of IOL rescue as presented in the above technique is the rapid visual recovery with significant visual improvement as early as 2 weeks post-operatively (see Fig. 2.). This is likely due to the benefit of avoiding entering the anterior chamber and inducing potential additional post-operative complications such as astigmatism, hypotony and corneal edema [9, 20]. 

A transscleral 4-point fixation technique with posterior threading of the suture through the IOL islets avoids many of the issues encountered with other forms of scleral fixation such as pupillary capture, pupillary block with secondary glaucoma, iris chaffing, iritis, uveitis-glaucoma-hyphaema [21, 22]. 

To avoid suture related complications associated with other sutures, many transscleral fixations techniques that limit suture friction have previously been employed such as scleral flaps/grooves or pockets, knotless z-suture techniques, burying of suture ends and adjustable buckle-slide sutures [8, 23, 24]. Gore-Tex is a non-absorbable, expanded polytetrafluoroethylene monofilament suture with strong tensile strength and which has been demonstrated to have excellent biocompatibility and resistance to degradation [23]. There were no cases of conjunctival erosion in a review of one year clinical outcomes of eyes undergoing combined PPV and scleral fixation of IOLs using Gore-Tex suture and we employed a similar technique where the Gore-Tex suture is tied on the scleral surface and covered at time of conjunctival closure [20]. 

The use of eight pars plana sclerotomies may seem excessive, and it is possible to perform the procedure through fewer wounds. A practical adaptation is to use 6 sclerotomies as 27-gauge forceps can fit through the 25-gauge ported sclerotomies alongside the suture. Our experience however was that this led to a longer procedure duration with more manipulation of the wounds which may pose increased risk of post-operative hypotony as opposed to the addition of 2 further 27-gauge sclerotomies. This was particularly noticeable when additional surgical manipulation was required such as cutting away capsular remnants and hardened residual lens matter.

Whilst long-term data is yet to be available, one-year outcomes of scleral fixation with Gore-Tex demonstrate an excellent safety profile with regards to suture erosion or breakage [20]. Patients were followed up to a minimum of six weeks postoperatively as per local protocol. Our novel anterior segment sparing, rescue re-suturing surgical technique, has a favourable post-operative complications profile with all instances resolving without surgical intervention with the exception of CME in one non-compliant patient. Previous evaluations of extended patient follow-up with Gore-Tex scleral fixation have showed that post-operative complications such as vitreous haemorrhage, CME, ocular hypertension/hypotony, hyphaema and corneal edema are diagnosed within ten days of surgery and resolve within first two post-operative months [20]. 

We found that our technique was applied successfully to a complex cohort of patients that exhibit similar risk factors previously described for IOL dislocation. These include trauma, zonular dehiscence, capsular bag contraction, pseudoexfoliation, connective tissue disorders, high myopia [6, 7, 25]. 

We acknowledge the lack of pre-operative and post-operative refractive data in this series and consequently a confounding factor in our final VA results may be refractive error. However, due to the length of time between cataract surgery and IOL dislocation, and particularly as most initial cataract surgery was performed at other units, it was not possible to acquire the intended refractive target and best corrected VA (BCVA) after cataract surgery. Our results therefore have relied on best measured acuity including PHVA which should eliminate the majority of the negative refractive element of VA assessment and provides a good estimate of BCVA. Thus, the effect of IOL rescue on refractive outcome relative to baseline remains unknown. Anecdotal evidence from patient enquiry however suggests that the majority of patients were comfortable using their pre-operative glasses suggesting that IOL rescue resulted in a similar effective lens position to prior to IOL dislocation.

In conclusion, we believe a Gore-Tex suture scleral re-fixation technique for dislocated 4-haptic IOLs is an effective, safe and practical approach which achieves excellent and rapid visual rehabilitation in the management of dislocated 4-piece IOLs.

### Electronic supplementary material

Below is the link to the electronic supplementary material.

Supplementary Material 1

## Data Availability

No datasets were generated or analysed during the current study.

## References

[1] Yamane S, Sato S, Maruyama-Inoue M, Kadonosono K. Flanged Intrascleral Intraocular Lens Fixation with Double-Needle Technique. Ophthalmology [Internet]. 2017;124:1136–42. 10.1016/j.ophtha.2017.03.036. 10.1016/j.ophtha.2017.03.036 28457613

[2] Kim SS, Smiddy WE, Feuer W, Shi W. Management of dislocated intraocular lenses. Ophthalmology [Internet]. 2008 [cited 2023 May 26];115:1699–704. https://pubmed.ncbi.nlm.nih.gov/18554720/. 10.1016/j.ophtha.2008.04.016 18554720

[3] Wagoner MD, Cox TA, Ariyasu RG, Jacobs DS, Karp CL. Intraocular lens implantation in the absence of capsular support: a report by the American Academy of Ophthalmology. Ophthalmology [Internet]. 2003 [cited 2023 May 26];110:840–59. https://pubmed.ncbi.nlm.nih.gov/12689913/. 10.1016/s0161-6420(02)02000-6 12689913

[4] Shin YI, Park UC (2020). Surgical Outcome of Refixation versus Exchange of dislocated intraocular Lens: a retrospective cohort study. J Clin Med [Internet].

[5] Chan CC, Crandall AS, Ahmed IIK. Ab externo scleral suture loop fixation for posterior chamber intraocular lens decentration: clinical results. J Cataract Refract Surg [Internet]. 2006 [cited 2023 May 26];32:121–8. https://pubmed.ncbi.nlm.nih.gov/16516790/. 10.1016/j.jcrs.2005.06.050 16516790

[6] Ascaso FJ, Huerva V, Grzybowski A, Epidemiology. Etiology, and Prevention of Late IOL-Capsular Bag Complex Dislocation: Review of the Literature. J Ophthalmol [Internet]. 2015 [cited 2023 May 26];2015. /pmc/articles/PMC4698990/. 10.1155/2015/805706 PMC4698990 26798506

[7] Mönestam E. Frequency of Intraocular Lens Dislocation and Pseudophacodonesis, 20 Years After Cataract Surgery - A Prospective Study. Am J Ophthalmol [Internet]. 2019 [cited 2023 May 30];198:215–22. https://pubmed.ncbi.nlm.nih.gov/30691613/. 10.1016/j.ajo.2018.10.020 30691613

[8] Zhang J, Tian J, Sun X, Yuan G, Williams GA. Closed Continuous-Loop Suture: A Novel Surgical Technique for Transscleral Fixation of Intraocular Lenses. Retina [Internet]. 2022 [cited 2023 May 21];42:2221–4. https://journals.lww.com/retinajournal/Fulltext/2022/11000/Closed_Continuous_Loop_Suture__A_Novel_Surgical.26.aspx. 10.1097/IAE.0000000000002644 31498288

[9] Nadal J, Kudsieh B, Casaroli-Marano RP. Scleral Fixation of Posteriorly Dislocated Intraocular Lenses by 23-Gauge Vitrectomy without Anterior Segment Approach. J Ophthalmol [Internet]. 2015 [cited 2023 May 30];2015. https://pubmed.ncbi.nlm.nih.gov/26294964/. 10.1155/2015/391619 PMC4532867 26294964

[10] Al-Halafi AM, Al-Harthi E, Al-Amro S, El-Asrar AA. Visual outcome and complications of pars plana vitrectomy for dislocated intraocular lenses. Saudi Journal of Ophthalmology [Internet]. 2011 [cited 2023 May 30];25:187. /pmc/articles/PMC3729642/. 10.1016/j.sjopt.2011.01.013 PMC3729642 23960921

[11] Ting DSJ, Tatham AJ, Donachie PHJ, Buchan JC (2023). The Royal College of Ophthalmologists’ National Ophthalmology Database study of cataract surgery: report 16, influence of remuneration model on choice of intraocular lens in the UK. Eye.

[12] Donachie PHJ, Barnes BL, Olaitan M, Sparrow JM, Buchan JC (2023). The Royal College of Ophthalmologists’ National Ophthalmology Database study of cataract surgery: Report 9, risk factors for posterior capsule opacification. Eye.

[13] Jabs DA, Nussenblatt RB, Rosenbaum JT, Atmaca LS, Becker MD, Brezin AP et al. Standardization of uveitis nomenclature for reporting clinical data. Results of the First International Workshop. Am J Ophthalmol [Internet]. 2005 [cited 2023 May 26];140:509–16. https://pubmed.ncbi.nlm.nih.gov/16196117/. 10.1016/j.ajo.2005.03.057 PMC8935739 16196117

[14] Day AC, Donachie PHJ, Sparrow JM, Johnston RL. The Royal College of Ophthalmologists’ National Ophthalmology Database study of cataract surgery: report 1, visual outcomes and complications. Eye (Lond) [Internet]. 2015 [cited 2023 Aug 2];29:552–60. https://pubmed.ncbi.nlm.nih.gov/25679413/. 10.1038/eye.2015.3 PMC4816350 25679413

[15] Moussa G, Bassilious K, Mathews N. A novel excel sheet conversion tool from Snellen fraction to LogMAR including ‘counting fingers’, ‘hand movement’, ‘light perception’ and ‘no light perception’ and focused review of literature of low visual acuity reference values. Acta Ophthalmol [Internet]. 2021;99:aos.14659. https://onlinelibrary.wiley.com/doi/10.1111/aos.14659. 10.1111/aos.14659 33326177

[16] Fan KC, Smiddy WE. Rescuing an Akreos 4-Point Haptic Intraocular Lens: A Novel Surgical Technique. RETINA [Internet]. 9000; https://journals.lww.com/retinajournal/Fulltext/9000/Rescuing_an_Akreos_4_Point_Haptic_Intraocular.95539.aspx. 10.1097/IAE.0000000000003159 33675331

[17] Hu X, Zhao B, Jin H. Intraocular Suture Looping Technique for Flapless Four-Point Refixation of Dislocated Intraocular Lenses. J Ophthalmol [Internet]. 2021 [cited 2023 May 26];2021. /pmc/articles/PMC7884157/. 10.1155/2021/6648777 PMC7884157 33628477

[18] Sarrafizadeh R, Ruby AJ, Hassan TS, Williams GA, Garretson BR, Trese MT et al. A comparison of visual results and complications in eyes with posterior chamber intraocular lens dislocation treated with pars plana vitrectomy and lens repositioning or lens exchange. Ophthalmology [Internet]. 2001 [cited 2023 May 26];108:82–9. https://pubmed.ncbi.nlm.nih.gov/11150269/. 10.1016/s0161-6420(00)00410-3 11150269

[19] Baba T, Nizawa T, Oshitari T, Yamamoto S. Comparisons of Visual and Surgical Outcomes after Reuse or Replacement of Dislocated in-the-Bag Intraocular Lens. J Ophthalmol [Internet]. 2018 [cited 2023 May 30];2018. https://pubmed.ncbi.nlm.nih.gov/29785302/. 10.1155/2018/7342917 PMC5896268 29785302

[20] Khan MA, Samara WA, Gerstenblith AT, Chiang A, Mehta S, Garg SJ, COMBINED PARS PLANA VITRECTOMY AND SCLERAL FIXATION OF AN INTRAOCULAR LENS USING GORE-TEX SUTURE. : One-Year Outcomes. Retina [Internet]. 2018 [cited 2023 May 26];38:1377–84. https://pubmed.ncbi.nlm.nih.gov/28492433/. 10.1097/IAE.0000000000001692 28492433

[21] Rahman R, Rosen PH. Pupillary capture after combined management of cataract and vitreoretinal pathology. J Cataract Refract Surg [Internet]. 2002 [cited 2023 May 26];28:1607–12. https://pubmed.ncbi.nlm.nih.gov/12231320/. 10.1016/s0886-3350(02)01212-9 12231320

[22] Ma DJ, Choi HJ, Kim MK, Wee WR. Clinical comparison of ciliary sulcus and pars plana locations for posterior chamber intraocular lens transscleral fixation. J Cataract Refract Surg [Internet]. 2011 [cited 2023 May 26];37:1439–46. https://pubmed.ncbi.nlm.nih.gov/21704487/. 10.1016/j.jcrs.2011.02.032 21704487

[23] Chen S, Yuan G, Zhu W, Shan T, Liu C, Zhang J. 8 – 0 Polypropylene Suture Looping and Overhand Knot: Transconjunctival Approach to Four-Point Scleral Fixation of an Akreos Adapt Intraocular Lens. Retina [Internet]. 2023 [cited 2023 May 26];43. https://pubmed.ncbi.nlm.nih.gov/32604345/. 10.1097/IAE.0000000000002873 32604345

[24] Vote BJ, Tranos P, Bunce C, Charteris DG, Da Cruz L. Long-term outcome of combined pars plana vitrectomy and scleral fixated sutured posterior chamber intraocular lens implantation. Am J Ophthalmol [Internet]. 2006 [cited 2023 May 29];141. https://pubmed.ncbi.nlm.nih.gov/16458685/. 10.1016/j.ajo.2005.09.012 16458685

[25] Tran THC, Zaier D, Proença J, Rouland JF. Posterior segment Intra-Ocular Implant (IOL) dislocation: Predisposing factors, surgical management, outcome analysis. J Fr Ophtalmol [Internet]. 2020 [cited 2023 May 30];43:1062–8. https://pubmed.ncbi.nlm.nih.gov/32811657/. 10.1016/j.jfo.2020.01.018 32811657

